# Filamentous Fungi as Excellent Industrial Strains: Development and Applications

**DOI:** 10.3390/jof10080541

**Published:** 2024-08-01

**Authors:** Mónica Gandía, Sandra Garrigues

**Affiliations:** 1 Preventive Medicine and Public Health, Food Science, Toxicology and Forensic Medicine Department, Faculty of Pharmacy and Food Sciences, Universitat de València, 46100 Burjassot, Valencia, Spain; monica.gandia@uv.es; 2 Food Biotechnology Department, Instituto de Agroquímica y Tecnología de Alimentos (IATA), Consejo Superior de Investigaciones Científicas (CSIC), 46980 Paterna, Valencia, Spain

## 1. Summary

Europe is transitioning towards a biologically based economy to reduce harmful and greenhouse emissions, promoting more sustainable industrial practices. In this context, filamentous fungi are well-known cell factories for bio-based approaches. Fungal biofactories are of great industrial interest due to their ability to produce high-value compounds, including organic acids, primary and secondary metabolites, enzymes, and other proteins, with applications in many industrial fields, such as food and feed, pulp and paper, detergent, textiles, packaging, pharmaceuticals, biochemicals, and biofuels. Industrial strains are commonly obtained by classical non-GMO strategies. However, in recent decades, genetic engineering, including genome editing, has significantly contributed to the development of efficient production strains with numerous potential applications at the industrial level. This Special Issue entitled ‘Filamentous fungi as excellent industrial strains: development and applications’ comprises seven manuscripts covering the use and improvement of filamentous fungi as sustainable biofactories for the production of industrially interesting compounds, particularly enzymes, as well as different methodologies to enhance such production. The main contents of this Special Issue can be summarized under the following topics.

## 2. Industrial Application of Filamentous Fungi for the Bioproduction of High-Value Compounds

The potential of filamentous fungi as efficient and sustainable cell factories for industrial purposes is undeniable. In particular, *Aspergillus oryzae* stands out for its strong capacity to synthesize secondary metabolites and produce proteins efficiently, even from agri-food wastes, as reviewed by Sun et al. [1]. The current market demand for natural compounds is driving the need for sustainable tools and strategies to improve their production. Metabolic engineering and synthetic biology are highly suitable approaches to enhance the ability of *A. oryzae* to grow and produce metabolites, as discussed in [1,2]. This filamentous fungus has proven capacity to produce industrially relevant metabolites such as kojic or citric acid, which are of interest in the cosmetic, pharmaceutical, and food industries. On the other hand, enzyme production is another outstanding aspect of *A. oryzae* as a cell factory, and, thanks to its efficient hydrolase system, it can also be used as a biofactory to process waste from the food and agricultural industries. These genetic and metabolic characteristics make it highly effective as a basis for the construction of cell factories in a bio-based economy.

In parallel, Vandelook and colleagues [3] focused on a less known fungal factory, *Trametes versicolor*, and investigated the use of orange peel extract (OPE) to enhance laccase production. *T. versicolor* is a wood-degrading fungus classified within the white-rot fungi, which are excellent wood decomposers. This is mediated by the secretion of Carbohydrate-Active EnZymes (CAZymes), which are powerful hydrolytic and oxidative enzymes, into the surrounding environment, such as laccases, which are involved in delignification reactions during wood degradation. Therefore, it is of significant interest to identify inducers for laccase-encoding genes that would result in improved laccase production. It was observed that the addition of OPE caused an upregulation of laccase gene expression without decreasing biomass production or fungal growth, which was the case with other chemical inducers tested. Since orange peels are an abundant waste product in the citrus fruit industry, their revalorization as an inducer of laccase production would provide a sustainable nutritional source within a circular economy framework.

Finally, another interesting industrial application of filamentous fungi is in the bioleaching process, a technique that uses microorganisms to extract metals from natural rocks or sediments. Using this technique, Vezzola and colleagues [4] studied the ability of a strain of *Aspergillus tubingensis* to extract metals from rocks simulating Martian regoliths. In their initial analysis, Vezzola and colleagues evaluated the strain’s ability to produce organic acids, followed by an assessment of its susceptibility to biomining processes. The *A. tubingensis* strain rapidly produced organic acids, lowering the pH of the culture medium and thereby creating suitable conditions for bioleaching. Furthermore, it produced siderophores, which can mobilize metals within rocks, enhancing metal extraction. Experiments with a Martian regolith simulant confirmed the strain’s effectiveness in the bioleaching process. Acidolysis and an increase in pH were observed, along with a decrease in organic acids, likely due to the formation of organometallic complexes and the decomposition of rock particles. These findings suggest that *A. tubingensis* is a promising candidate for bioleaching and open new research pathways for the sustainable acquisition of resources in space exploration.

## 3. Strategies for the Development of Fungal Biofactories

Recombinant DNA technology has made it possible to improve fungal strains by adding, deleting, or modifying specific genes in a more precise manner than classical mutagenesis. Techniques such as genetic engineering and genome editing are contributing to the development of industrial production strains. Nevertheless, there is still room for further strain improvement.

The review by Salazar-Cerezo et al. [5] focused on the description of classical and recent methods, tools, and technologies used for the development of fungal production strains with the potential to be applied on an industrial scale. Among the classical methods, physical and chemical mutagenesis, adaptive evolution, protoplast fusion, and genome shuffling strategies were discussed. Additionally, the main transformation methods for the modification of fungal strains through genetic engineering were described, which were protoplast-mediated transformation (PMT), electroporation, and *Agrobacterium tumefaciens*-mediated transformation (ATMT). Particularly, ATMT has the advantage of transforming fungi simply by mixing *Agrobacterium* cells with spores, mycelia, and even fruiting bodies of the target fungus without the need for removing the cell wall, contrary to PMT. Therefore, Yoon et al. [6] established an efficient ATMT protocol to genetically manipulate *Phialemonium inflatum* for the first time, which is a useful aquatic fungus known for its ability to mineralize lignin and decompose polycyclic aromatic hydrocarbons (PAHs). This optimized transformation system will enable functional analyses to study genes of interest in *P. inflatum* and its exploitation for the production of interesting metabolites with high industrial value.

Salazar-Cerezo and colleagues also reviewed genetic engineering-based methods for the rational modification of filamentous fungi [5]. In this regard, distinct synthetic biology and genome editing approaches were discussed. Additionally, Yang et al. [2] reviewed the advancements in tools for the genetic modification of *A. oryzae*. These tools ranged from conventional DNA manipulation to the most sophisticated modular cloning strategies, such as Gateway or Gibson assembly. Additionally, genome-editing technologies, including zinc finger nucleases (ZFNs), transcription-like activator effector nucleases (TALENs), and CRISPR/Cas9, were discussed for their suitability for gene function studies on *A. oryzae*.

Finally, Salazar-Cerezo et al. [5] reviewed different genetic engineering approaches to improve the industrial potential of fungi, which included strategies for gene downregulation or inactivation, i.e., gene deletion, point mutations, or RNA interference (RNAi), and gene up-regulation, i.e., promoter swaps, or an increase in the gene copy number. These strategies were also shown in Yang et al. and Sun et al. for enhanced protein and secondary metabolite production in *A. oryzae* through the generation of strains with modified genes resulting in reduced protease activity or improved secretion pathways, for example [1,2].

## 4. The Role of -Omics Technologies in the Development of Fungal Biofactories for Industrial Applications

Advances in obtaining and analyzing complete fungal genomes and gene expression patterns, as well as the proteome, have led to the development of integrative -omics tools and a deep understanding of fungal biology. This knowledge has also contributed to the rapid advancement of synthetic and systems biology strategies, broadening insights into fungal development. In this context, Li et al. explored six evolutionarily diverse fungal species—*Aspergillus niger*, *Aspergillus nidulans*, *Penicillium subrubescens*, *Trichoderma reesei*, *Phanerochaete chrysosporium*, and *Dichomitus squalens*—and their CAZymes at the genomic and transcriptomic level, providing valuable insight into the diverse mechanisms employed by fungi to degrade plant biomass [7].

Results reveal significant diversity in CAZyme numbers and expression across the six fungal species. This diversity directly impacts the ability of fungi to utilize various polysaccharides as growth substrates. Fungi with a higher presence of these enzymes exhibited a greater capacity to grow on a broad spectrum of polysaccharides, whereas fungi with a lower CAZyme gene content showed more restricted growth capacity. In addition, the study identified sugar-specific induction patterns for CAZyme-encoding genes, indicating intricate regulatory mechanisms governing polysaccharide degradation in different fungi. Generally, Eurotiomycetes displayed a greater diversity of these genes, allowing them to grow on a wider range of polysaccharides compared to other species. The wood-degrading Basidiomycete fungi showed different gene expression profiles compared to the Ascomycetes, likely due to distinct adaptations for growing on complex polysaccharides in their natural environments. Understanding these differences may lead to the development of more efficient enzyme mixtures and the use of metabolic engineering to optimize fungal strains for specific industrial applications, such as plant biomass degradation.

## 5. Conclusions

In general, the papers presented in this Special Issue reflect the efforts of the scientific community to provide filamentous fungal strains with improved properties. These strains were developed to be applied at the industrial level and represent development trends in the area of fungal biofactories within the framework of sustainability.

We greatly appreciate the contributions of each author and reviewer that made this Special Issue possible. We hope that more studies will emerge in the second edition of the Special Issue ‘Filamentous Fungi as Excellent Industrial Strains: Development and Applications’ in the near future.

## References

[1] Sun Z., Wu Y., Long S., Feng S., Jia X., Hu Y., Ma M., Liu J., Zeng B. (2024). *Aspergillus oryzae* as a Cell Factory: Research and Applications in Industrial Production. J. Fungi.

[2] Yang H., Song C., Liu C., Wang P. (2024). Synthetic Biology Tools for Engineering *Aspergillus oryzae*. J. Fungi.

[3] Vandelook S., Bassleer B., Elsacker E., Peeters E. (2024). Effects of Orange Peel Extract on Laccase Activity and Gene Expression in *Trametes versicolor*. J. Fungi.

[4] Vezzola M., Tosi S., Doria E., Bonazzi M., Alvaro M., Sanfilippo A. (2023). Interaction between a Martian Regolith Simulant and Fungal Organic Acids in the Biomining Perspective. J. Fungi.

[5] Salazar-Cerezo S., de Vries R.P., Garrigues S. (2023). Strategies for the Development of Industrial Fungal Producing Strains. J. Fungi.

[6] Yoon J., Kim Y., Kim S., Jeong H., Park J., Jeong M.H., Park S., Jo M., An S., Park J. (2023). *Agrobacterium tumefaciens*-Mediated Transformation of the Aquatic Fungus *Phialemonium inflatum* FBCC-F1546. J. Fungi.

[7] Li J., Wiebenga A., Lipzen A., Ng V., Tejomurthula S., Zhang Y., Grigoriev I.V., Peng M., de Vries R.P. (2023). Comparative Genomics and Transcriptomics Analyses Reveal Divergent Plant Biomass-Degrading Strategies in Fungi. J. Fungi.

